# Keratolysis Associated with Methamphetamine Use – Incidental Diagnosis of Corneal Melt in a Patient with Acute Methamphetamine Intoxication

**DOI:** 10.5811/cpcem.2020.3.43981

**Published:** 2020-06-15

**Authors:** Jagdipak S. Heer, Sean Heavey, Daniel Quesada, Phillip Aguìñiga-Navarrete, Madison B. Garrett, Kieron Barkataki

**Affiliations:** * Kern Medical Center, Department of Emergency Medicine, Bakersfield, California; † LAC+ USC Medical Center, Department of Emergency Medicine, Los Angeles, California

**Keywords:** corneal ulceration, keratolysis, methamphetamine

## Abstract

**Case Presentation:**

A 38-year-old male presented to the emergency department with methamphetamine-induced agitation. Physical exam showed clouding of the left cornea, with gelatinous appearance and associated conjunctivitis, consistent with corneal melt, or keratolysis.

**Discussion:**

Keratolysis is dissolution of the corneal stroma that can lead to corneal ulceration and vision loss. Smoking stimulants has been shown to be associated with this pattern of ocular injury, although this is a relatively rare presentation. Acute keratolysis is a unique complication of methamphetamine preparation and ingestion via smoking that can lead to corneal ulceration and loss of vision.

## CASE PRESENTATION

A 38-year-old male with a history of drug use was brought to the emergency department (ED) by law enforcement for evaluation of chest pain and acute agitation. The patient had a known history of methamphetamine use, and a urine drug screen in the ED was positive for methamphetamines. Complete medical history and initial physical exam were unable to be performed due to patient’s agitation. Cardiac work-up, including chest radiograph, electrocardiograph, and troponin, was unremarkable. The patient was found to have elevated creatine kinase, concerning for rhabdomyolysis, but otherwise normal chemistries. He received benzodiazepines for his agitation and combativeness and was started on intravenous fluids for rhabdomyolysis.

Once he was calm, further physical examination was notable for clouding of the left cornea, with a gelatinous appearance overlying the left pupil and associated conjunctivitis (Image). The right pupil was normal. Formal visual acuity was not performed as patient was unable to cooperate with the exam. After consulting with ophthalmology, it was determined the patient had keratolysis, likely associated with methamphetamine use. He was started on maxitrol and prednisone acetate drops with subsequent admission to internal medicine for management of methamphetamine-induced rhabdomyolysis.

## DISCUSSION

Ocular injuries including corneal ulceration and ocular foreign bodies account for 1–2% of all ED visits.^1^ Corneal ulceration is a feared complication of missed ocular injuries and can result in visual impairment. Keratolysis, or “corneal melting,” is a phenomenon well described in ophthalmology literature. It is defined as progressive dissolution of the corneal stroma, which if untreated can cause corneal perforation and vision loss. It is most commonly caused by autoimmune destruction, infection, or inflammation.^2^ In rare cases, aerosolized and inhaled stimulant use, including crack cocaine and methamphetamine, has been associated with keratitis and keratolysis.^3^,^4^ Although the exact mechanism of ocular injury is unknown, there are a number of factors related to drug use that may cause direct ocular injury including preparation of methamphetamine, smoking and thermal injuries, exposure to caustic chemicals used to produce methamphetamine, or exposure to impurities or additives used to dilute, or “cut” the methamphetamine.

Acute methamphetamine intoxication is a common problem seen in EDs, particularly in western states where methamphetamine use is highest.^5^ Acutely intoxicated patients may be aggressive toward staff, unable to communicate symptoms, or unable to participate with physical examination. Because of these challenges, missed diagnoses resulting in incomplete or delayed care are common.^6^,^7^ This case re-emphasizes the importance of careful and complete physical examinations in acutely agitated patients or patients presenting with clinical intoxication.

**CPC-EM Capsule**

What do we already know about this clinical entity?

Corneal keratolysis is progressive dissolution of the corneal stroma, causing corneal ulceration and vision loss caused by infection, inflammation, or chemical injury.

What is the major impact of the image(s)?

Keratolysis is an uncommon ocular injury. Recognition and prompt treatment may help prevent further damage and vision loss.

How might this improve emergency medicine practice?

This image will aid in identification of corneal keratolysis and serve as a reminder of its possible association with smoking and manufacturing methamphetamine.

## Figures and Tables

**Image:**
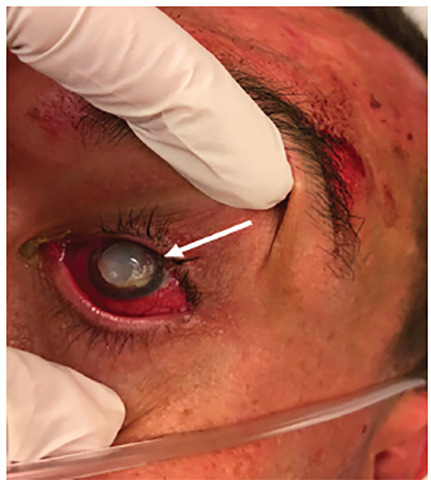
Left eye showing clouding of the cornea, consistent with the diagnosis of corneal melting (arrow).

## References

[1] Nash EA, Margo CE (1998). Patterns of emergency department visits for disorders of the eye and ocular adnexa. Arch Ophthalmol.

[2] Hossain P (2012). The corneal melting point. Eye (Lond).

[3] Poulsen EJ, Mannis MJ, Chang SD (1996). Keratitis in methamphetamine abusers. Cornea.

[4] Ghosheh FR, Ehlers JP, Ayres BD (2007). Corneal ulcers associated with aerosolized crack cocaine use. Cornea.

[5] Artigiani EE, Hsu MH, McCandlish D (2018). Methamphetamine: A Regional Drug Crisis.

[6] Tucci V, Siever K, Matorin A (2015). Down the rabbit hole: emergency department medical clearance of patients with psychiatric or behavioral emergencies. Emerg Med Clin North Am.

[7] Klein LR, Cole JB, Driver BE (2018). Unsuspected critical illness among emergency department patients presenting for acute alcohol intoxication. Ann Emerg Med.

